# Innate Immune Responses to Chimpanzee Adenovirus Vector 155 Vaccination in Mice and Monkeys

**DOI:** 10.3389/fimmu.2020.579872

**Published:** 2020-11-30

**Authors:** Catherine Collignon, Vanesa Bol, Aurélie Chalon, Naveen Surendran, Sandra Morel, Robert A. van den Berg, Stefania Capone, Viviane Bechtold, Stéphane T. Temmerman

**Affiliations:** ^1^ Preclinical R&D, GSK, Rixensart, Belgium; ^2^ Discovery Performance Unit, GSK, Rockville, MD, United States; ^3^ Preclinical R&D, ReiThera Srl, Rome, Italy

**Keywords:** innate immunity, chimpanzee adenovirus, adenovirus vector, vaccine, mice, monkeys

## Abstract

Replication-deficient chimpanzee adenovirus (ChAd) vectors represent an attractive vaccine platform and are thus employed as vaccine candidates against several infectious diseases. Since inducing effective immunity depends on the interplay between innate and adaptive immunity, a deeper understanding of innate immune responses elicited by intramuscularly injected ChAd vectors in tissues can advance the platform’s development. Using different candidate vaccines based on the Group C ChAd type 155 (ChAd155) vector, we characterized early immune responses in injected muscles and draining lymph nodes (dLNs) from mice, and complemented these analyses by evaluating cytokine responses and gene expression patterns in peripheral blood from ChAd155-injected macaques. In mice, vector DNA levels gradually decreased post-immunization, but local transgene mRNA expression exhibited two transient peaks [at 6 h and Day (D)5], which were most obvious in dLNs. This dynamic pattern was mirrored by the innate responses in tissues, which developed as early as 1–3 h (cytokines/chemokines) or D1 (immune cells) post-vaccination. They were characterized by a CCL2- and CXCL9/10-dominated chemokine profile, peaking at 6 h (with CXCL10/CCL2 signals also detectable in serum) and D7, and clear immune-cell infiltration peaks at D1/D2 and D6/D7. Experiments with a green fluorescent protein-expressing ChAd155 vector revealed infiltrating hematopoietic cell subsets at the injection site. Cell infiltrates comprised mostly monocytes in muscles, and NK cells, T cells, dendritic cells, monocytes, and B cells in dLNs. Similar bimodal dynamics were observed in whole-blood gene signatures in macaques: most of the 17 enriched immune/innate signaling pathways were significantly upregulated at D1 and D7 and downregulated at D3, and clustering analysis revealed stronger similarities between D1 and D7 signatures versus the D3 signature. Serum cytokine responses (CXCL10, IL1Ra, and low-level IFN-α) in macaques were predominantly observed at D1. Altogether, the early immune responses exhibited bimodal kinetics with transient peaks at D1/D2 and D6/D7, mostly with an IFN-associated signature, and these features were remarkably consistent across most analyzed parameters in murine tissues and macaque blood. These compelling observations reveal a novel aspect of the dynamics of innate immunity induced by ChAd155-vectored vaccines, and contribute to ongoing research to better understand how adenovectors can promote vaccine-induced immunity.

## Introduction

Replication-incompetent adenovirus (Ad) vectors, such as those based on the well-studied human Ad serotype 5 (Ad5), are employed as vaccine delivery vehicles due to their high manufacturing efficiency, versatility, and capacity to accommodate large transgenes (1). They also enable efficient transduction of dividing and non-dividing cells without chromosomal integration (2). Owing to their lower seroprevalence and thus decreased vector neutralization in humans as compared to human Ad5, chimpanzee Ad (ChAd) vectors represent an attractive vaccine platform (3–5). ChAd-vectored candidate vaccines against infectious diseases such as malaria, Ebola, and RSV have shown favorable immunogenicity and tolerability in humans (6–11). These vaccines drive robust intracellular antigen expression, thus promoting a CD8^+^ T-cell-biased response, but were also shown to induce antigen-specific CD4^+^ T-cell responses and antibodies. The latter responses can be enhanced by modifying the transgene-coding sequence to favor extracellular protein release. These properties have guided the selection of this platform for several vaccine candidates with a ChAd serotype 155 [ChAd155 (12, 13)] backbone. These vaccines include the pediatric RSV vaccine ChAd155-RSV (7) currently in Phase II development (NCT02927873), as well as the rabies vaccine ChAd155-RG and the therapeutic hepatitis B vaccine ChAd155-hIi-HBV, both in Phase I development (NCT04019444 and NCT03866187, respectively).

The development of persisting transgene-specific immunity requires activation of the innate arm of the immune system, for which the Ad vector’s hexon protein may act as an intrinsic adjuvant (14). Upon intramuscular (i.m.) vaccination, the interplay between diverse innate signals―such as pattern-recognition receptor activation, immune-cell recruitment to the injection site, and cytokine production―shapes adaptive responses to the transgene (15). Innate immune recognition of the vector is thus necessary to trigger its self-adjuvanticity, but some murine and *in vitro* studies suggest that certain innate cues can also dampen Ad infection efficiency and antigen expression, either directly by killing infected cells, or indirectly *via* cytokine production (16–18). This balance between immune suppression and stimulation appears to be defined by levels of vector-induced effector cells (NK cells, neutrophils, monocytes/macrophages), and expression of interferon (IFN) signaling–related genes in the draining lymph node (dLN) (15–19). In addition, the innate response quality/magnitude is shaped by the vaccine delivery route, Ad serotype, and host, as well as by the anatomical site of the response (17–20). The latter influence is exemplified by the difference between blood and dLN expression levels of certain cell-associated transcripts seen after subcutaneous administration of Ad vectors in mice (17). Understanding how Ad vectors interact with the innate immune system is thus essential for optimal vaccine development. Several aspects of innate immunity to Ad vectors, administered *via* various delivery routes, have been unraveled in the context of vaccination, gene therapy, or infection (15–17, 21–24). However, for i.m. injection, the preferred route for human vaccines, there is a need for a more comprehensive understanding of the early events occurring not only in the dLN or serum, but also at the first point of entry, the muscle.

Here, we characterize the innate immune response to intramuscularly delivered ChAd155 vectors combined with different antigens [i.e., ChAd155-RSV, ChAd155-RG, or the green-fluorescent-protein (GFP)–expressing vector ChAd155-GFP] in two animal models. We first studied tissue-specific vector DNA levels, transgene expression, cytokines/chemokines expression, and immune-cell infiltration in the injected muscle and dLNs from C57BL/6 mice, and then explored cytokine/chemokine and gene expression in peripheral blood from the translationally more relevant non-human primate (NHP) model. We observed a remarkable concordance between the two models, with a bias toward IFN-associated responses, and, intriguingly, a bimodal dynamic pattern. By characterizing, for the first time, the early immune mechanisms induced by ChAd155-vectored vaccines, this study will contribute to our understanding of the adaptive immunity data that are currently emerging from the clinical trials evaluating these vaccines. Additionally, our data will help explain the immunogenicity profiles of adenoviral vaccines at large, and advance the vaccine platform.

## Materials and Methods

### ChAd155-Vectored Vaccines

The ChAd155-RSV and ChAd155-RG candidate vaccines are based on the replication-defective (E1/E4-deleted) ChAd155 vector (7, 25). Vector construction and production have been described (12, 13, 25), and a GFP-expressing ChAd155 vector (ChAd155-GFP) was developed using similar procedures. In each of the investigated vaccines, the transcription of the transgene is driven by the human cytomegalovirus (hCMV) promoter, and the bovine growth hormone poly-adenylation signal sequence is downstream of the transgene stop codon. ChAd155-RSV encodes a secreted form of hRSV fusion (F) protein deleted of the transmembrane and cytoplasmic regions (F0ΔTM), and a fusion of nucleocapsid (N) and anti-termination (M2-1) proteins (7). ChAd155-RG encodes rabies virus glycoprotein (25).

### Animals and Immunizations

The studies in mice and NHP were conducted at the AAALAC-accredited facilities of GSK (Rixensart, Belgium) and Aptuit (Verona, Italy), respectively. Animal husbandry and experiments were ethically reviewed and carried out in accordance with the European Directive 2010/63/EU and the GlaxoSmithKline Biologicals S.A. Policy on the Care, Welfare and Treatment of animals. The study in NHP was conducted in accordance with the Italian legislation, under approval of the facility’s Committee on Animal Research and Ethics, and under authorization issued by the Italian Ministry of Health (Italian Ministry of Health Authorization n. 984/2015-PR; Aptuit Internal code no. 50000).

**Figure 1:**
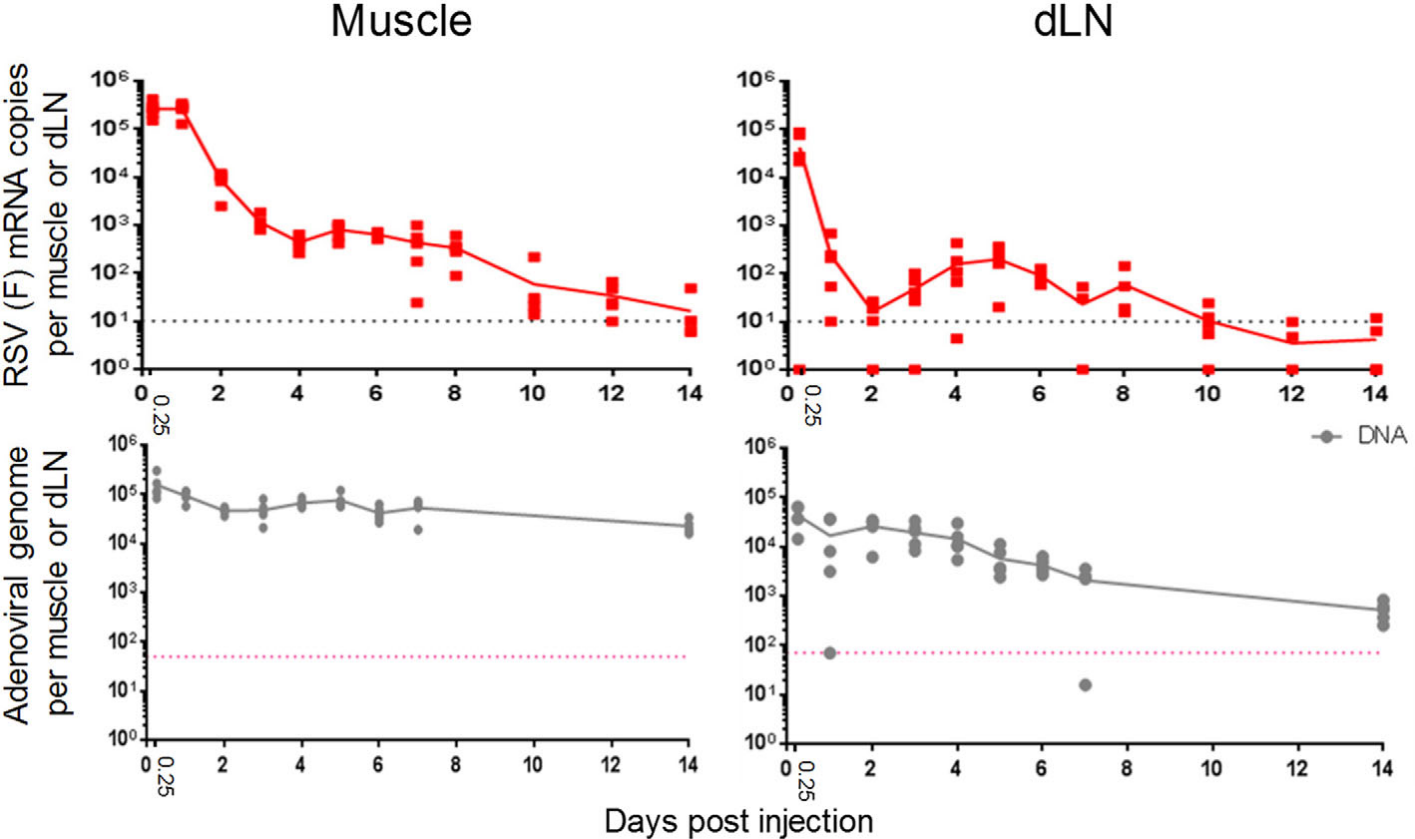
Different kinetics of transgene mRNA expression *versus* viral vector DNA levels in mice. Expression levels of RSV fusion (F) mRNA and ChAd155 viral DNA levels (top and bottom panels, respectively) detected in murine muscles (left) and draining lymph nodes (dLN; right) are shown. Mice (N = 5/time point) were injected intramuscularly with ChAd155-RSV (5 × 10^8^ vp) or buffer (control) at Day 0. Injections were performed either unilaterally as one full dose for mRNA quantification, or bilaterally as two half doses for DNA quantification. Depending on the time point, graphs represent data from two or three (mRNA) or one or two (DNA) independent experiments. Solid lines represent mean expression levels; symbols represent means or single values per animal. Dotted lines represent the lower limits of quantitation.

**Figure 2:**
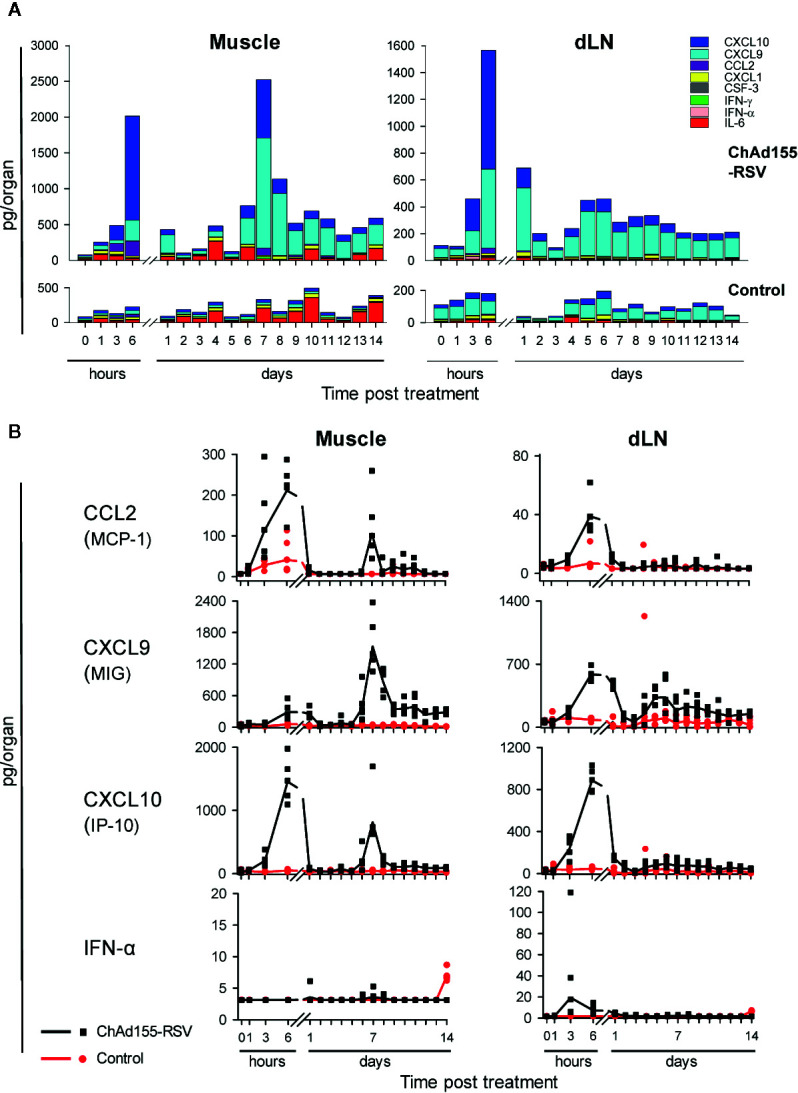
Local cytokine responses in mice are dominated by CXCL10 and CXCL9. Cytokine responses in homogenates of the injected muscles and draining lymph nodes (dLN) collected from mice treated with ChAd155-RSV (10^8^ vp) or buffer (“Control”) are shown (n = 5/group/time point). Injections (10 µl) were performed unilaterally in the left gastrocnemius muscle at Day 0. Tissues were collected before immunization (0 h), and after immunization at 1, 3, and 6 h, and then daily from 24 h (Day 1) through Day 14. Graphs represent data from a single experiment. Cytokine levels (expressed in pg/organ) were detected in homogenate supernatants of the indicated tissues collected from the injected side of each animal. Geometric mean concentrations are presented as cumulative bars **(A)** or lines **(B)**. Each symbol in **(B)** represents one animal.

**Figure 3:**
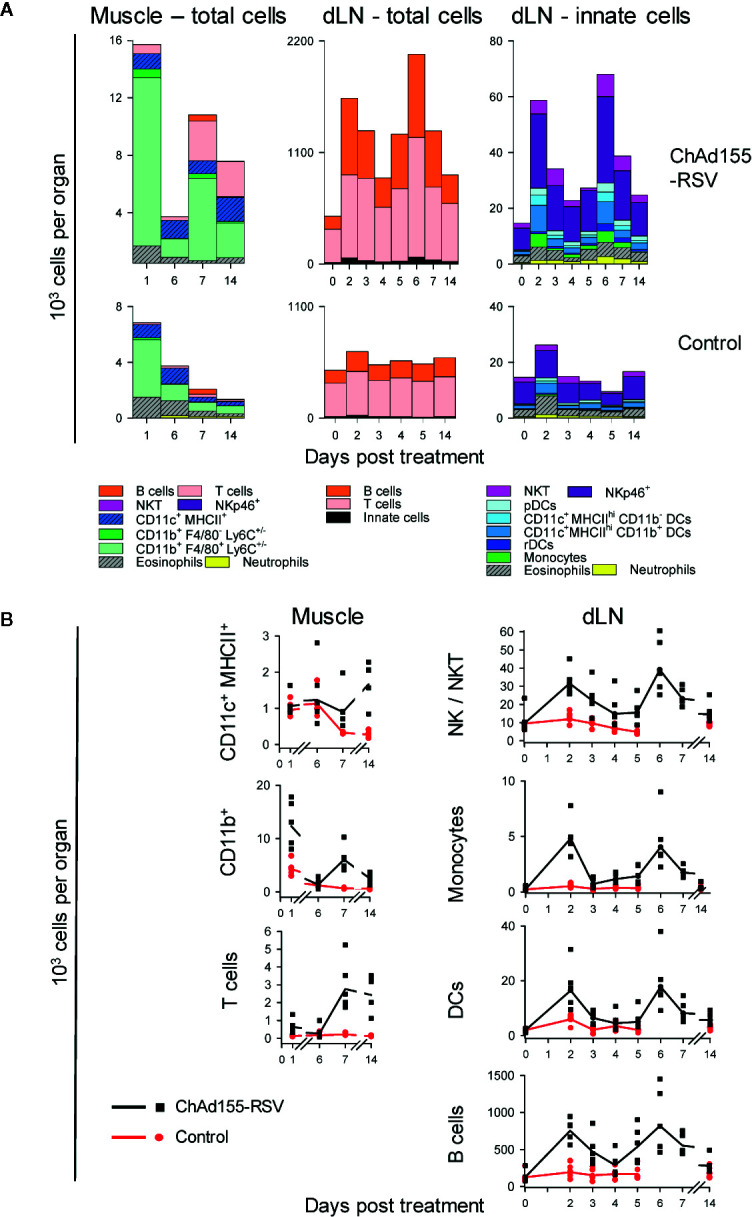
Kinetics of immune cell infiltration in mice. The composition of immune cell infiltrates in muscles and draining lymph nodes (dLNs) collected from mice (n = 5/group/time point) immunized with ChAd155 RSV (10^8^ vp) or phosphate-buffered saline (“Control”) are shown. Injections were performed bilaterally as two half doses (10 µl each) in both gastrocnemius muscles at Day 0. Injected muscles were collected after immunization (Days 1, 6, 7, and 14); dLNs were collected both before (0 h) and after immunization (Days 2–7 and 14; note the discontinuity in time points on x-axes). All cells recovered from muscles and 10^6^ cells of the total recovered dLN cells were stained and analyzed by flow cytometry; values represent both the cell count per tissue and the number of total events detected by FACS (i.e., CD45^+^ living muscle cells and total living dLN cells). Geometric means of number of cells per tissue are presented as cumulative bars **(A)** or lines **(B)**. Each symbol in **(B)** represents one animal. Graphs represent data from single experiments performed for each tissue.

**Figure 4:**
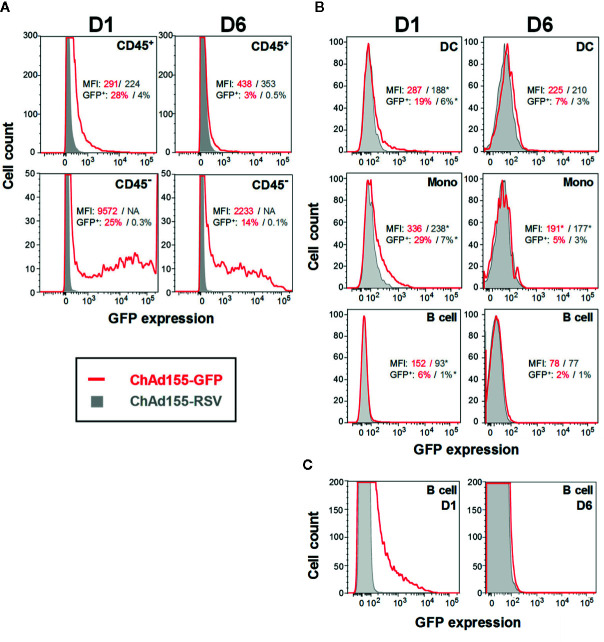
Antigen expression and distribution in local cell populations after vaccination. Histograms representing green fluorescent protein (GFP)-expressing cells detected in either hematopoietic (CD45^+^) or non-hematopoietic (CD45^−^) cell subsets in muscle cell suspensions **(A)**, or in immune-cell subsets in draining lymph node (dLN) cell suspensions **(B)** are shown. Given the loss of detail with the scaling of **(B)** which was needed to accommodate the different population sizes, a detail-zoom of the dLN B cells is presented in **(C)**. Mice were immunized with ChAd155-GFP or the control vaccine ChAd155-RSV (both at 5 × 10^9^ vp; typically n = 5/time point). Injections were performed in both gastrocnemius muscles at 2.5 × 10^9^ vp/muscle. Tissues were collected at 1 or 6 days post-immunization (D1, D6). Experiments were performed once (muscle) or in duplicate (dLN). Graphs represent data generated for a single representative animal. Geometric means of the mean fluorescence intensity (“MFI”) values and of the frequencies of GFP-expressing (“GFP^+^”) cells measured in the ChAd155-GFP group (red font) and ChAd155-RSV group (grey font) are indicated in each graph; asterisks denote values that were calculated from only 1 or 2 samples (as MFI was not measured for samples with <50 positive events). DC, dendritic cells; including plasmacytoid DCs, resident DCs and migratory CD11b^+/−^ DCs. Mono, monocytes. B cell, CD19^+^ cell populations.

Female 6- to 8-week-old C57BL/6 mice (Harlan Horst) were randomly allocated across the study groups (n = 5/time point/group/experiment). They were immunized by i.m. injection (*gastrocnemius* muscle) with ChAd155-RSV, ChAd155-GFP, or placebo control (each at 10 µl/muscle) at Day (D)0. Injections were administered either unilaterally as one full dose in the left muscle [**Figure 1** (mRNA), **Figure 2**], or bilaterally as two half doses [**Figure 1** (DNA), **Figures 3** and **4**). ChAd155-RSV vector doses per animal were 5.0 × 10^8^ vp in **Figure 1** and 1.0×10^8^ vp in **Figures 2** and **3**. To ensure adequate flow cytometric analyses, and based on published data (17), a higher dose of ChAd155-RSV or ChAd155-GFP (i.e., 5.0 × 10^9^ vp) was used in **Figure 4**. The controls were phosphate-buffered saline (PBS) in **Figure 3**, and A195 buffer (26) in all other experiments. In determinations of immune response kinetics (**Figures 2** and **3**), an additional control group of untreated mice was used to characterize baseline (D0) responses. The number of repeats performed for each experiment in mice is indicated in the individual analysis descriptions (see below) and figure legends. Blood samples, injected muscles and iliac dLNs were collected at different time points as indicated in the figures. The animals had free access to water and a maintenance diet. Nesting material was included in the cages, and social housing was applied.

Six 3-year-old cynomolgus macaques (*Macaca fascicularis*) weighing 3.17–4.05 kg were obtained from LCL-Cynologics IBL House; Port Louis, Mauritius. They received 300 µl ChAd155-RG (5 × 10^10^ vp; within the range of doses intended to be used in humans) administered as a single injection in the deltoid muscle at D0, as described (25). In that study, blood samples for assessment of humoral responses were taken at prevaccination and from weeks 2 to 48 post first vaccination, and virus-neutralizing antibody titers in sera were measured as described (25). Blood samples for whole-blood gene expression and serum cytokine analyses were collected five days before immunization (“Pre”), and at D1, D3, and D7 post-immunization. For ethical reasons, these experiments were performed only once. The animals were housed in communicating steel cages (maintained at 21°C –23°C; RH 45%–65%; lighting between 06:00 and 18:00 h with 30 min dawn-sunset light system), with access to environmental enrichment devices. No clinical signs were noted either before or after immunization (25).

### Anti-RSV F Antibody Response in Mice

Anti-RSV F immunoglobulin (Ig)G concentrations in sera from mice immunized with ChAd155-RSV (10^8^ vp/animal) at D0 were measured using an ELISA developed in-house with a cutoff of 50 ELISA units (EU)/ml. Blood samples were collected daily up to D14 (n = 5/time point), and then, in a separate experiment, at D14, D21, D34, D56, and D77 (n = 10/time point).

### Monitoring of RSV Transgene Expression in Mice

Total RNA was isolated by homogenizing murine muscle and dLN tissues in Tripure (Sigma) used in quantities adapted according to the tissue’s volume, and extracted with phenol/chloroform, then treated using the RNeasy Mini kit (Qiagen). The RNeasy column was treated with DNase I (Qiagen). RNA yields were quantified using a NanoDrop 2000c spectrophotometer. The total RNA profile was analyzed using the RNA6000 Nano kit with the 2100 Bioanalyzer for muscle tissue, and the High Sensitivity RNA Screen Tape kit in Tape Station 4200 (Agilent) for dLNs. RSV transcripts were quantified by qPCR using TaqMan Gene Expression Master Mix Plus on a ViiA7 sequence detection system (Applied Biosystems). The primers and probe encoding the F0ΔTM sequence in ChAd155-RSV were: 5′-cgagcccatcatcaacttct-3′ forward primer; 5′-caggctctggttgatcttctc-3′ reverse primer; and 5′-6FAM-cgagttcgacgccagcatca-BHQ1-3′ probe. For each sample, 200 ng (muscle) or 50 ng (dLN) RNA were reverse transcribed in 20 μl with SuperScript III (Invitrogen), and volumes of 2 μl (muscle) or 4 μl (dLN) were analyzed. Expression was measured against a dilution curve of ChAd155-RSV (5 × 10^7^–5 copies). Amplifications were performed in duplicate. Taqman Gene Expression assay was used to detect *Tfrc* expression (Mm00441941_m1; Applied Biosystems). Depending on the time point, experiments were performed in triplicate (6 h, D2, D6) or duplicate (other time points).

### Monitoring of Viral DNA in Mice

For adenoviral genome DNA quantification, tissues were lysed (3 h, 56°C) in 40 μl Proteinase K (Qiagen), using 160 μl ATL buffer per whole dLN or per 20-mg muscle tissue, then 200 μl of the homogenized lysates were incubated (10 min, 70°C) with 200 μl AL buffer (all Qiagen). Mice genomic DNA and adenoviral DNA were co-extracted with the QIAamp DNA Mini purification kit (Qiagen) according to the manufacturer’s instructions, and quantified using a NanoDrop 2000c spectrophotometer (Thermo Fisher Scientific). DNA quantification by qPCR was performed on 10 μl per sample (corresponding to 10% of the total volume of available DNA) using TaqMan Gene Expression Master Mix on a ViiA7 sequence detection system (Applied Biosystems), against a ChAd155-RSV dilution curve (10^8^–10 copies). Primers and probe (IDT) were directed at the hCMV promoter sequence in ChAd155-RSV [hCMV1 primers left (cagtacatcaatgggcgtggatag) and right (attttggaaagtcccgttgattttg); hCMV1 probe (6FAM-cggggatttccaagtctccacc-BHQ1)]. Taqman Copy Number Reference assay (Applied Biosystems, ThermoFisher Scientific cat. #4458366) was used to detect the housekeeping gene (HKG) transferrin receptor (*Tfrc*). Depending on the time point, experiments were performed in duplicate (6 h, D2, D6) or only once (other time points).

### Cytokine Levels in Mice and Monkeys

Cytokine levels were measured in murine and monkey sera, and in murine tissues. Tissue samples dissected from muscles and dLN were individually homogenized, and cytokine levels were measured in tissue-homogenate supernatants as described previously (27, 28). Due to the high number of time points analyzed, the experiments in mice were performed only once. Analyzed cytokines included IFN-γ, IFN-α, IL-6, CXCL1, CSF-3, CCL2, CXCL9, and CXCL10 in mice, and 30 cytokines (IFN-γ, IFN-α, CXCL-10, CXCL11, CXCL9, IL-1β, IL1Ra, IL-7, IL-8, IL-6, IL-18, CCL2, CCL4, CCL3, CXCL12, CCL11, CXCL13, CSF-3, CSF-1, IL-15, IL-10, IL-12p70, sCD40L, IL-4, IL-5, IL-13, IL-17A, IL-2, IL-23, and TNF-α) in monkeys. ELISA kits were used to measure levels of IFN-γ (R&D Systems; in mice), IFN-α (PBL Assay Science; in mice/NHP), or CXCL10 (R&D systems human kit; in NHP). All other cytokines were measured by multiplex assays, using Luminex xMAP (Millipore) for murine samples (including sera, and muscle- and dLN-tissue homogenates), and ProcartaPlex (Invitrogen) for monkey sera. For calculation of geometric mean concentrations (GMCs) or medians, concentrations below the assay’s lower limit of quantitation (LLOQ) were assigned a value of either half the manufacturer’s LLOQ (for the NHP multiplex assay and IFN-α ELISA), or equal to the manufacturer’s limit of detection (LOD; for the NHP CXCL10 ELISA, and the mouse multiplex assay and IFN-α ELISA). In NHP, statistically significant differences between post- and pre-vaccination levels were calculated using an analysis of variance (ANOVA) model with repeated measures including the time point as fixed effect. The model was fitted on log_10_-transformed cytokine concentrations. A compound symmetry covariance structure was selected, and homogeneity of variances was assumed. The level of significance was set at *P* < 0.05.

### Murine Muscle and dLN Cell Phenotyping Using Flow Cytometry

From each mouse, both injected muscles were homogenized in GentleMACS in a solution of DMEM containing 1% FCS, 100 µg/ml Dnase I (Roche) and 0.1 U/ml Liberase TM (Roche) at 37°C. After 1 h, the enzymatic reaction was stopped by addition of a cold solution of DMEM containing 1% FCS and 10 mM EDTA. After centrifugation and filtration, cells recovered were subjected to a Percoll density gradient. All cells subsequently recovered were transferred into 96 V-bottom wells and washed in FACS buffer (PBS containing 2 mM EDTA and 2% FCS). Both dLNs were treated individually by mechanical dissociation in 2 ml RPMI medium + 2% FCS. After addition of Dnase I and Liberase (at 150 µg/ml and 0.13 U/ml, respectively; Roche), and incubation (30 min; room temperature; under agitation), digestion was stopped by addition of 10 mM EDTA and incubation on ice. The dLN cells were filtered (100 µM nylon cell strainer, BD Biosciences), washed and resuspended in PBS containing 2 mM EDTA and 2% FCS before counting. All cells recovered from the muscle, and 10^6^ dLN cells, were treated with CD16/32 (clone 2.4G2; BD Pharmingen) and CD16.2 (clone 9e9; Biolegend) antibodies (10 min, 4°C) to block the Fc receptor, and stained (30 min, 4°C) with the anti-mouse antibodies described below.

For kinetics characterization (**Figure 3**), cells extracted from muscles were stained with anti-CD90.2-FITC, anti-CD45-PE, anti-SiglecF-PECF594, anti-CD43-BV510, anti-Ly6C-BV605, and anti-Ly6G-BV711 from BD Pharmingen; anti-F4/80-APC from Miltenyi Biotec; anti-CD335-PE/Dazzle 594, anti-CD19-BV421, and anti-CD11b-BV785 from Biolegend; anti-CD11c-PE-Cy7 and anti-MHCII-Alexa Fluor 700 from eBiosciences; and Live/Dead Near-IR from Invitrogen. The dLNs were stained with anti-NK1.1-PE, anti-CD43-BV500, anti-Ly6C-BV605, and anti-Ly6G-BV711 from BD Pharmingen; anti-SiglecH-APC, anti-CD19-BV421, and anti-CD11b-BV785 from Biolegend; anti-TCRb-PercpCy5.5, anti-CD11c-PE-Cy7, and anti-MHCII-Alexa Fluor 700 from eBiosciences; and Live/Dead Near-IR from Invitrogen. Due to the high number of time points analyzed, these experiments were performed only once.

In experiments using ChAd155-GFP (**Figure 4**), cells recovered from muscles were stained with anti-CD45-APC and Live/Dead Near-IR from Invitrogen, and dLN cells were stained with the same dLN antibody panel as described above. These experiments were performed once (muscle) or in duplicate (dLN).

Fluorescent events were acquired using an LSR2 flow cytometer (BD Biosciences), and analyzed using FlowJo software v9 (Tree Star); see **Figure S1** for the gating strategies applied for each tissue. For statistical analyses in the experiments with ChAd155-GFP, the following subsets were pooled: neutrophils and eosinophils (“granulocytes”), plasmacytoid DCs, resident DCs and migratory CD11b^+/−^ DCs (“DCs”), and TCRβ^+^ NK1.1^+^ cells and NK1.1^+^ cells (“total NK cells”).

### Whole Blood Gene Expression in NHP Using Nanostring

Total RNA was isolated from whole blood from NHP that was collected in PAXgene Blood RNA tubes (PreAnalytiX), using the RNeasy RNA purification kit and the BioRobot MDx system (both Qiagen, Valencia, CA, USA) according to the manufacturer’s guidelines. RNA concentrations were measured by ND-1000 spectrophotometer (NanoDrop Technologies). RNA integrity numbers (RINs) were determined using the Agilent 2100 Bioanalyzer and Agilent RNA 6000 Nano kit (Agilent Technologies). RNA with RIN >7 was included in the analysis. Total RNA samples were analyzed using a pre-designed gene expression code-set targeting 730 immune-related NHP genes (nCounter NHP Immunology Panel, NanoString Technologies). Probeset-target RNA hybridization reactions, using 50 ng total RNA/reaction, were performed according to the manufacturer’s protocol. Purified probeset-target RNA complexes from each reaction were processed and immobilized on nCounter cartridges using the nCounter Flex Prep Station, and transcripts were quantified on the nCounter Digital Analyzer GEN 2 (all NanoString Technologies). Raw data was analyzed using Nanostring nSolver 4.0 software and the Advanced Analysis Module 2.0 plugin, as described [(29, 30) and **Supplementary Methods**]. For pathway scoring, scores were calculated as the first principal component of the normalized expression (raw counts) of the genes included in a dedicated pathway. For this analysis, at least one co-variate was chosen against which the scores were plotted (i.e., the time points), thus reflecting any factor(s) emerging as the main driver(s) of variability in the gene expression of that particular gene set.

For the gene-set analysis, pathways enriched by the DEGs were determined by the directed significance for a covariate, as determined by the cumulative evidence of DEGs in a pathway, and calculated as the square root of the mean squared t-statistics of the genes. The presence of over- or under-expressed genes in a pathway was determined by the directed global significance, taking the direction of the t-statistics sign into account (31).

### Whole Blood Gene Expression in NHP Using qPCR

To validate Nanostring data, expression of 84 immune-related genes was assessed by qPCR. RNA quality and quantity were assessed as described above. Reverse transcription was performed using the RT^2^ First Strand Kit (Qiagen; 400 ng RNA/reaction). Transcription levels of 89 immune-related genes and the HKGs GAPDH, LOC709186 and RPL13A were measured by qPCR using the RT^2^ Profiler Rhesus Macaque Innate & Adaptive Immune Responses arrays (Qiagen) and a ViiA7 real-time cycler. Each qPCR reaction was qualified and validated in a specific range of threshold cycle (Ct) values. For values higher than the LOQ (Ct = 32), the value was replaced by “LOQ + 1”. Geometric means of the Ct of the three HKG and means of the Ct for each duplicated target gene were calculated, with the following normalization for each target gene: ΔCt = geomean Ct_HKG_ − mean Ct_target gene_. The impact of vaccination on the mRNA levels was expressed in ΔΔCt values, representing the relative quantification of the ΔCt values at D1, D3, or D7 over the ΔCt at pre-vaccination (D-5), by calculating ΔΔCt = ΔCt post − ΔCt pre. Fold changes (FCs) were calculated as 2^ΔΔCt^. Genes with FC > |2.0| were considered differentially expressed. Genes with discrepancies between the *Macaca mullata*-specific primes and *Macaca fascularis* sequences were excluded from the analyses.

## Results

### Transgene Expression and Viral Vector DNA Levels Follow Different Kinetics in Murine Tissues

Immunogenicity of a single i.m. immunization with ChAd155-RSV (10^8^ vp/animal) in C57BL/6 mice was confirmed by assessing RSV F-specific antibody responses in sera. Data from two separate experiments revealed detectable responses from D8, in 40% of the animals (**Figure S2**). All animals exhibited a response by D10. Geometric mean concentrations increased up to D12 and then plateaued through D77, with responses persisting in all mice.

We then initiated our main analysis by evaluating the local events following i.m. ChAd155 vaccination. The kinetics of RSV F mRNA expression and viral vector DNA persistence were characterized in tissues from ChAd155-RSV-treated mice, using mock-treated mice as control group. For both parameters, levels in muscles and dLNs were measured at 6 h and D1 post-immunization, then daily or every other day up to D14 (mRNA), or daily up to D7 and at D14 (DNA; **Figure 1**).

In the muscle, RSV F mRNA levels were maintained at levels exceeding 10^5^ copies between 6 and 24 h, but then decreased by 1-log by D2, and by 2-log by D4. Levels remained relatively constant through D8, to gradually decrease to near-undetectable levels by D14. By contrast, viral DNA levels were relatively stable, though with an overall 1-log decrease, up to D14.

Unlike the early (≤ 24 h) gene expression in the muscle, mRNA expression in the dLN showed already from 6 h a rapid and pronounced (4-log) decrease, to reach negligible levels at D1-D2. This was followed by a 1-log increase until D5, and gradual contraction to non-quantifiable levels from D10 onward. As in the muscle, DNA levels only gradually decreased (by 2-log) and remained detectable up to at least D14.

The kinetics of DNA levels may be consistent with the ≥7-week persistence of ChAd155 DNA levels in muscles and dLNs of ChAd155-RG injected rats (25), and suggest that the decline in transgene expression seen here in both tissues within the first few days post-immunization was not caused by limiting levels of viral particles, but rather by regulation at the level of mRNA. We next characterized local immune responses to ChAd155 vectors.

### Early Cytokine Responses to ChAd155 Are Dominated by IFN-Related Chemokines and Follow Bimodal Kinetics

To elucidate the vector’s ability to promote early cytokine production following i.m. vaccination, we compared the cytokine/chemokine responses in injected muscles and dLNs from ChAd155-RSV-immunized mice with those in mock (buffer)-treated mice, up to two weeks after a single injection at D0.

At the muscle injection site, cytokine responses were initiated as early as 1 h post-treatment, and reflected at 6 h a CCL2-, CXCL9-, and CXCL10-dominated peak (**Figures 2A, B**, *left*). After contracting to baseline at D1 or D2, this response was followed by a second peak of these cytokines at D7, this time dominated by CXCL9 and coinciding with a low IFN-γ peak (**Figure S3A**, *left*). This bimodal kinetic pattern could also be discerned, though less obvious, in the dLN (**Figures 2A, B** and **Figure S3A**; *right*). In this tissue, a minor IFN-α signal at 3 h was followed at 6 h by a low IFN-γ response and higher CCL2, CXCL9, and CXCL10 responses, all of which had contracted by D2 or D3. For CXCL9, this first response was followed by a second (lower) increase around D5–D7, which was still detectable at D14. The minor IFN-α response observed in both tissues from control mice at D14 was considered an anomaly. The late time point, and the fact that it was neither detected in vaccinated mice at D14, nor at any of the preceding time points in the control mice, suggest that it was unlikely to be related to the placebo injection.

In serum, the vaccine elicited at 6 h only CCL2 and CXCL10 responses (**Figure S3B**). Neither the tissues nor the serum exhibited substantial vaccine-induced responses of the neutrophil-associated chemokines CXCL1 and CSF-3, or of the pro-inflammatory cytokine IL-6, at any of the time points measured.

Given the roles of the detected chemokines in recruitment of immune cells such as lymphocytes (IFN-inducible chemokines CXCL9 and CXCL10), monocytes (CXCL10 and CCL2), and DCs (CCL2) (32–34), we next investigated immune-cell infiltration in the same tissues.

### Immune Cell Infiltration Coincides With Chemokine Responses

To characterize the dynamics of local cell recruitment, we analyzed the composition of immune-cell infiltrates in muscles and dLNs from ChAd155-RSV-treated and mock-treated mice up to D14, using flow cytometry (see **Figure S1** for tissue-specific gating strategies).

In the muscle, recruited cells consisted predominantly of (CD11b^+^ F4/80^+^ Ly6C^+/−^) monocytes or monocyte-derived macrophages (**Figure 3A**, *left*). Recruitment of these cells occurred in two separate peaks, at D1 and D7 (cell counts at D1/D6/D7: 12/1/6 × 10^3^ cells/muscle), coinciding with the two CCL2 peaks in this tissue (see **Figure 2**). This suggested that locally produced CCL2 induced recruitment of mainly patrolling (CD11b^+^ F4/80^+^ Ly6C^−^) monocytes to the muscle injection site. In addition, increased numbers of CD11c^+^ MHCII^+^ DCs (D14) and T cells (D7, D14) were detected in ChAd155-injected muscles, while NK (NKT/NKp46^+^) cells were not detected in either group at any of the four time points tested.

In the dLN, total cell numbers had tripled from baseline levels at D2, then contracted at D4, and more than doubled again at D6 (D2/D4/D6 cell counts: ~1,600/900/2,100 × 10^3^ per dLN; **Figure 3A**, *middle*). Both peaks were characterized by a strong lymphocyte component, as T and B cells constituted 97% of all dLN cells, with the second peak possibly representing the initiation of the adaptive response. Deeper analysis of the dLN innate-cell compartment demonstrated that NK cells constituted approximately half of the recruited innate cells at D2 (**Figure 3A**, *right*), suggesting a link with the CXCL9/10 responses (see **Figure 2**). Slightly lower responses of DCs and monocytes were also observed. Neutrophil recruitment was negligible, consistent with the absence of CXCL1 or CSF-3 responses.

Thus, the two temporally distinct peaks in chemokine production were reflected in the cell recruitment patterns, with a 1-day delay in the dLN possibly reflecting the time-span the chemokine signal required to promote such spatial recruitment. The response was dominated by CD11b^+^ cells in the muscle and NK, T, B cells and DCs in the dLN. This prompted us to determine the relative contributions of these cells to antigen expression.

### ChAd155 Infects Mostly B Cells and Monocytes in the dLN

To complement chemokine-production and cell-migration patterns, we next determined the cellular tropism of a GFP-encoding ChAd155 vector at the response peaks (D1, D6) in tissues, by identifying the cell subsets preferentially targeted by the vector. ChAd155-RSV was used as control, to track potential interference due to cell auto-fluorescence (a common limitation of *in vivo* GFP detection).

Upon CD45 staining of muscle cells, both the non-hematopoietic (CD45^−^) and hematopoietic (CD45^+^) subsets expressed GFP (**Figure 4A**), suggesting that muscle-resident non-immune cells, likely myocytes, as well as infiltrating blood cells are targets of ChAd155. Though the gated cells did not represent the full cell population in the muscle due to experimental procedures (see Materials and Methods), mean fluorescence intensity (MFI) was higher in the non-hematopoietic subset [D1 geometric means: 9572 (CD45^−^) vs. 291 (CD45^+^)]. Coinciding with the kinetics of the main infiltrating subset in the muscle (CD11b^+^ F4/80^+^ Ly6C^+/−^ cells; **Figure 3**), GFP^+^ CD45^+^ cell frequencies declined sharply from D1 to D6, while fluorescence signals remained relatively stable [D1/D6 geometric means: 28%/3%; 291/438 (MFI)]. A more moderate decline in GFP^+^ cell frequencies was seen in CD45^−^ cells (D1/D6 geometric means: 25%/14%).

In the dLN, cells with potential APC functionalities including DCs and monocytes (**Figure 4B**), as well as B cells (**Figures 4B, C**) also exhibited GFP expression at both time points, aligned with the kinetics of immune cell infiltration (**Figure 3**). In addition, the D6 signals in B cells (**Figures 4B, C**) could be aligned with the initiation of the antibody response detected from D8 in serum (**Figure S2**). For other cell types, levels detected in the ChAd155-GFP group were ambiguous (granulocytes), or relatively low and/or similar to controls (NK and T cells; data not shown). While our analyses did not allow discrimination between signals emanating from transduced cells or from phagocytosed infected cells, the late time points and detected intensities suggested that the former process was responsible for the bulk of these signals.

### IFN Pathway Activation and Gene Expression Kinetics in NHP Blood Are Consistent With Chemokine Signatures in Murine Tissues

Previous studies illustrated discrepancies in Ad vector-induced innate cytokine profiles between NHP and mice, potentially related to differences in receptor binding, i.e., to CD46, which is lacking in mice, vs. to coxsackievirus and Ad receptor (CAR) (20). We therefore aimed to bridge the local analyses in mice with systemic (serum) responses in NHP. Cytokine levels and immune-related gene expression were evaluated at pre-vaccination (D-5) and D1, D3, and D7 post-vaccination, in serum from macaques previously injected with ChAd155-RG at D0. The humoral and cell-mediated adaptive immunogenicity of a single dose of this vaccine was previously demonstrated in the same animals, by characterizing the rabies virus-neutralizing antibody responses and rabies-specific IFN-γ–expressing T-cell responses in peripheral blood (25).

**Figure 5:**
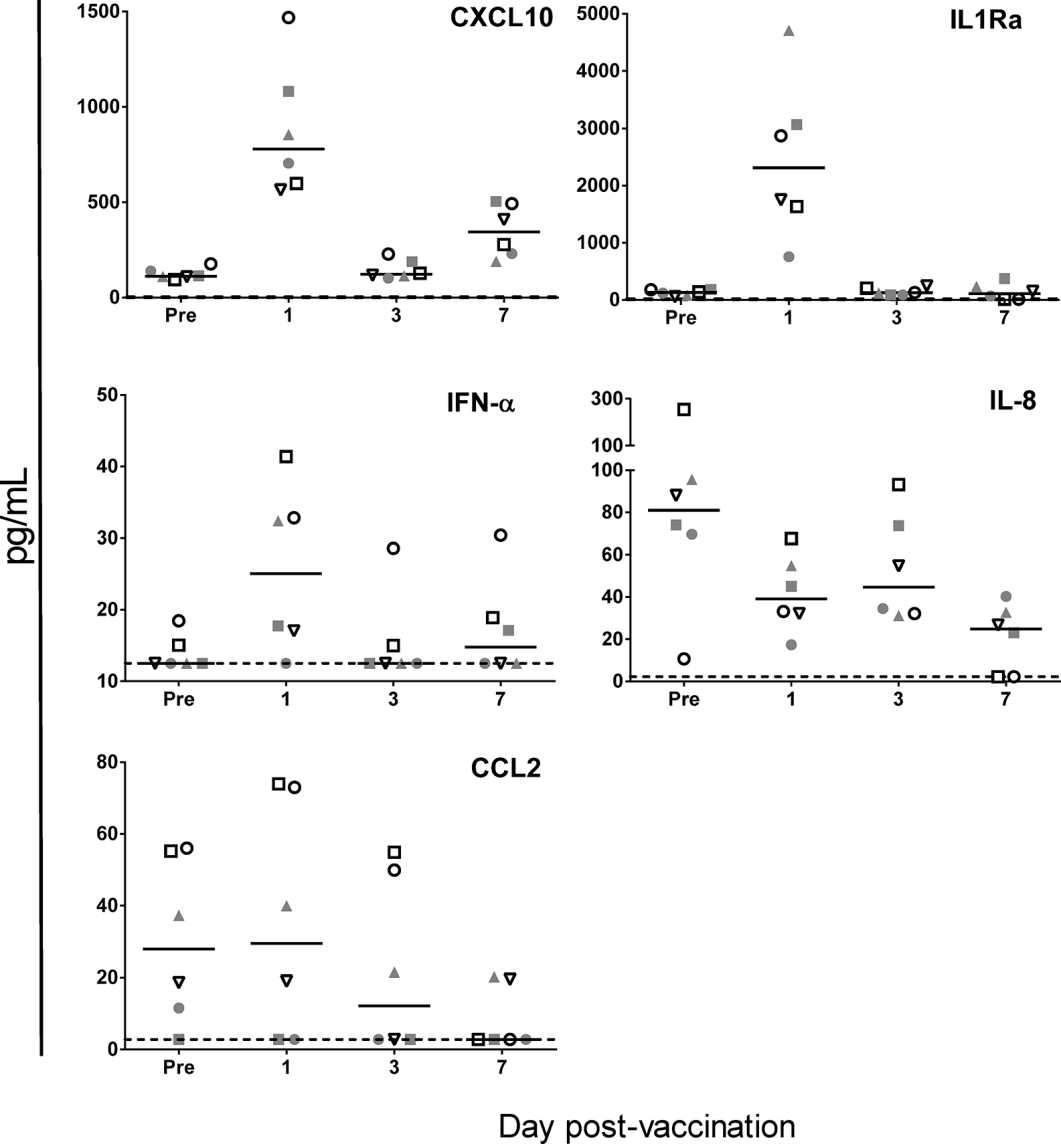
ChAd155-vectored rabies vaccine elicits few serum cytokine responses in macaques. Cytokine levels were measured at pre-vaccination (“Pre”, Day-5) and 1, 3, and 7 days post- vaccination in serum collected from six macaques injected intramuscularly with ChAd155-RG rabies vaccine (5 × 10^10^ vp) at Day 0. Graphs shown [CXCL10, IFN-α (both ELISA), IL1Ra, IL-8, and CCL2 (multiplex assay)] represent the only five analytes found to be modulated by vaccination, out of the panel of 30 immune-related cytokines, growth factors or activation markers tested. Each symbol represents the same animal across the graphs. Solid lines represent medians; dashed lines represent limits of detection applied for statistical calculations. An ANOVA model of the log_10_-transformed geometric mean concentrations revealed significant changes as compared to prevaccination for CXCL10 at Days 1 and 7, IL-1RA at Day 1 (all *P* < 0.001) and IL-8 at Day 7 (*P* < 0.01), as well as a borderline significant change (*P* = 0.05) for IFN-α at Day 1.

Of the panel of 30 immune-related cytokines, growth factors or activation markers, only nine cytokines were detected, and only five were modulated by vaccination (**Figure 5**). As compared to pre-vaccination baseline, we detected slightly increased IFN-α levels at D1, and stronger increases of both CXCL10, at D1 and D7, and of the anti-inflammatory marker IL1Ra, at D1. Statistical analysis by ANOVA revealed that these increases were borderline significant for IFN-α and significant for CXCL10 and IL1Ra (see figure legend for details). We also detected significantly decreased levels of the neutrophil chemoattractant IL-8 at D7, and a statistically non-significant trend toward decreased CCL2 levels at D3 and D7. Thus, while the CXCL10 and IFN responses corresponded with murine data, the absence of CCL2 increases did not, though comparisons may be obfuscated by the fewer time points investigated in the NHP model. Baseline CXCL12, CCL11, CCL4, and IL-7 levels were unchanged after vaccination (data not shown).

We then evaluated the dynamic regulation and signatures of whole-blood transcriptomic responses by differential gene expression analyses (*P*-value < 0.05), performed with NanoString technology on a 730-gene panel. Consistent with the response kinetics in murine tissues, numbers of differentially expressed genes (DEG) revealed a bimodal kinetic pattern, with higher numbers of upregulated [log_2_ fold-change (FC) >1] DEGs at D1 and D7 as compared to D3 (upregulated/total DEGs D1, D3, and D7: 61/318, 3/149, and 36/285, respectively; **Table S1**).

**Figure 6:**
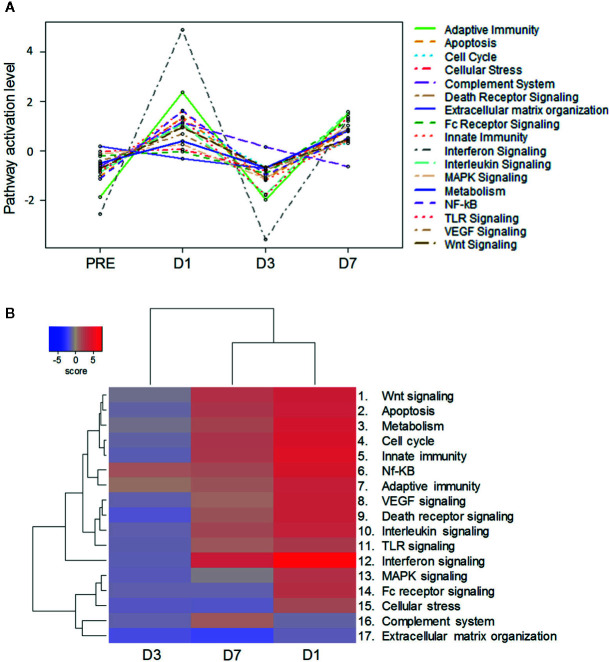
Bimodal kinetic pattern of gene signatures in ChAd155-RG-immunized macaques. Whole blood was collected at pre-vaccination (“PRE”) and 1, 3 and 7 days post vaccination (D1, D3, D7) from six macaques injected intramuscularly with ChAd155-RG rabies vaccine (5 × 10^10^ vp) at Day 0. Modulation of gene expression across timepoints was analyzed through pathway scoring **(A)** and gene set analysis **(B)**, with both methods leading to analysis of pathway modulation at the different timepoints. Significantly enriched immune-related pathways are listed at the right side of each panel (see **Table S2** for gene lists per pathway). **(A)** For pathway scoring, principal component (PC) analysis was performed for each sample using a linear combination (weighted average) of its gene expression values, weighing specific genes to capture the greatest possible variability in the data. The first PC reflects any factor(s) emerging as main driver(s) of variability in gene expression of that particular gene set. **(B)** Gene expression values were expressed as log_2_ fold-changes over baseline (*P*-value < 0.05). Differential expression and pathway enrichment analyses were performed by direct global significance analyses using NanoString nSolver 4.0 with Advanced Analysis 2.0 plugin (730-gene panel).

Gene-set enrichment analyses revealed 17 enriched pathways associated with innate and adaptive immunity (see **Table S2** for genes annotated into each pathway). Pathway scores obtained using principal component analysis followed a similar dynamic pattern, with scores for most modulated pathways increasing from baseline to D1, then decreasing to levels at or below baseline at D3, followed by a second increase at D7 (**Figure 6A**). Consequently, gene-set and clustering analyses based on log_2_ fold-changes over baseline (*P*-value < 0.05) revealed a greater similarity between the D1 and D7 expression profiles as compared to the D3 profile (**Figure 6B**). The highest enrichment scores were observed for the IFN signaling-related pathway, both at D1 and D7. Interestingly, most genes associated with the NF-kB pathway (regulating apoptosis, inflammatory responses and cell growth) and the adaptive immunity pathway were upregulated at all three time points, indicating these processes were less impacted by the mechanisms underlying the downregulation of the other pathways. Five pathways followed different kinetics as they were not upregulated at either D1 (complement system- or extracellular matrix organization-associated pathways) and/or D7 (pathways associated with MAPK signaling, Fc receptor signaling, cellular stress, or extracellular matrix organization), but they were all downregulated at D3.

Subsequent qPCR validation performed for 84 genes identified 12 genes that were differentially expressed (log_2 _FC >1) on at least one time point post-vaccination (**Table S3**). Trends and kinetics of expression levels (typically D1 > D7 > D3) and the bias toward IFN-associated genes (9/12 of DEGs) were consistent with those seen for the broader (Nanostring) gene panel. Overall, the predominance of IFN-associated protein and gene signatures, and the bimodal kinetic pattern of these transcriptomic responses corroborated the patterns seen in murine local tissues.

## Discussion

Innate immunity is required to orchestrate the sequence of immunological events resulting in the desired transgene-specific adaptive response (15). A deeper understanding of the kinetics and nature of innate immunity in tissues elicited after i.m. vaccination is thus essential for future improvement of adenoviral vaccine design. We have characterized for the first time the innate mechanisms underlying the adaptive immunogenicity of vaccines based on the clinically relevant ChAd155 vector. Using ChAd155 vectors expressing different antigens, we characterized the early immune responses in injected muscles and dLNs from mice, and compared these data with the protein and gene-expression patterns in blood from macaques. There was a remarkable concordance in innate immune patterns between both animal models, and three key observations were made. First, from 6 or 24 h post-immunization, ChAd155-vectored vaccines induced local tissue-specific innate cell population changes and cytokine production, which, along with local transgene expression and blood transcriptomic responses, mostly followed a bimodal temporal profile. Second, innate immune activation was in both animal models dominated by IFN-associated signatures. Last, the vector induced only low-level responses of either inflammatory cytokines in blood (both models) or tissues (mice), or inflammatory cell-infiltrates in tissues in mice.

**Figure 7:**
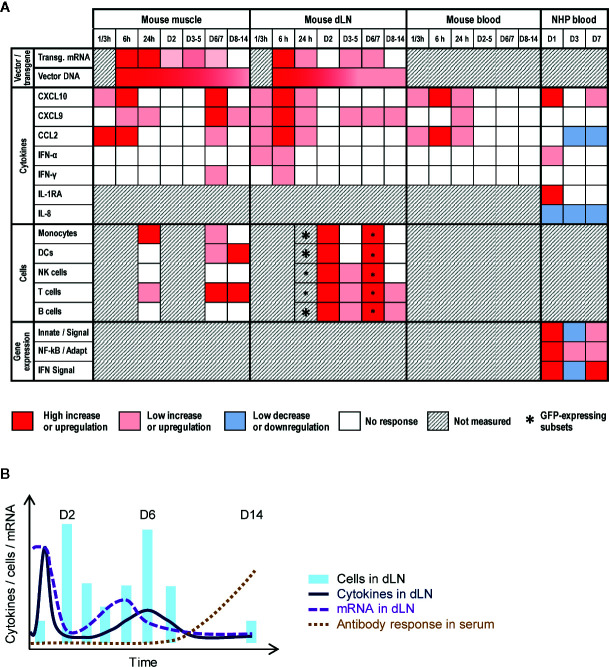
Conceptual data integration. **(A)** Conceptual heat map representing the response levels in the injected muscle or draining lymph node (dLN) from mice, or in peripheral blood from mice or non-human primates (NHP) after intramuscular immunization with ChAd155-vectored vaccines. Blood responses were measured in serum (cytokines, in both animal models) or whole blood (gene expression). Colors represent the qualitative response intensity as compared to pre-vaccination levels, as indicated in the color key below the figure. Asterisk sizes correspond to relative response levels. Indicated gene expression refers to the following pathways in **Figure 6B**: “Innate/signal”, innate immunity signaling pathways (nos. 1–5 and 8–11); “NF-KB/Adapt”, NF-kB and adaptive immunity pathways (nos. 6 and 7); and “IFN signal”, interferon signaling pathway (no. 12). **(B)** Diagram representing hypothetical model of transgene mRNA expression and immune response in the dLN elicited after immunization. Transgene mRNA expression peaks shortly after injection, but is rapidly dampened, likely by the first wave of cytokine/chemokine release and subsequent cell mobilization (D2). Upon contraction of this first innate response wave around D4–D5, a residual reservoir of ChAd155-infected cells instigates a reactivation of viral gene expression, and consequently also of innate immune responses (D6–D7). These responses are then followed by adaptive immunity, including the antigen-specific antibody response which was detectable in the serum from D8.

Based on our dataset (see overview in **Figure 7A**), we propose the following hypothetical mechanism. In the injected muscle, the vaccine vector infected hematopoietic cells [generally present in very low quantities before injection (35–37)] and muscle cells, and both subsets probably contributed to transgene mRNA expression at 6 h. This led to production of danger signals, stimulating muscle-resident innate sentinels to engulf the vector and secrete cytokines (e.g., CXCL10, and CCL2) within a few hours. In turn, this promoted monocyte/macrophage chemotaxis to the muscle (D1)―likely aligned with the concurrent upregulation of innate/IFN transcriptomic pathways in (NHP) blood―resulting in further local cytokine secretion, removal of free antigen and cellular debris, and reduction of infected cells. Collectively this could instigate IFN-mediated downregulation of transgene transcription (D2–D4).

In the dLN, the transgene mRNA levels detected at 6 h suggested that antigen may have localized to this tissue in different capacities, such as in free-flowing viral particles or as cargo within phagocytotic cells. The kinetics of the immune response (i.e., cellular migration, cytokine responses) and viral mRNA expression suggest a temporal interplay between host and vector (**Figure 7B**). Indeed, apart from the effects as a result of the drainage described above, secretion of CXCL9/10, and, to a lesser extent, CCL2, IFN-γ, and IFN-α, may also be responsible for the D1/D2 cell influx (supposedly followed by antigen uptake in subcapsular sinuses). This concurred with a sharp decline in mRNA levels from 6 h to D2, which was likely due to regulation at the mRNA level considering the coinciding persistent DNA levels. Indeed, since the hCMV promoter used in ChAd155 vectors to drive transgene transcription (12, 13), is reportedly inhibited by IFN-γ (38), this steep drop in mRNA levels may be linked to the early cytokine signals. Additionally, as DC and monocyte levels subsequently decreased more quickly from the dLN relative to NK-cell levels, the reduced transcription may also be mediated *via* NK-cell action, possibly activated by IFN-α (16). These patterns in murine tissues were consistent with the downregulation of innate pathways in monkey blood at D3. Of note, considering that in ChAd-vectored vaccines, the noninfectious particles vastly outnumber infectious particles (39), lack of mRNA production following the uptake of noninfectious particles by APCs will have skewed the mRNA/DNA balance strongly to the side of DNA (**Figure 1**).

Remarkably, a second, lower-level immune activation in tissues was seen at D6/D7. This was possibly linked to residual expression of (likely biologically active) mRNA remaining in myocytes around D2/D3. This mRNA could subsequently have accumulated in the absence of concurrent robust innate and cytokine responses. The transient D6/D7 innate immune activation could be in keeping with a possible reversion of the putative IFN-γ–mediated inhibition of expression (38) after the first innate response peak had receded. The response in tissues was aligned with the second upregulation of innate/IFN-related pathways at D7 in (NHP) blood, and we hypothesize that this secondary response contained higher frequencies of antigen-specific T cells as compared to the D1 activation. Though T-cell CXCR3 expression was not analyzed, this sequence of immune events would fit a previously described model of CXCR3-dependent immunosurveillance and elimination of infected cells by CD8 T cells (40, 41). In this model, immune cells, such as monocytes or DCs, upregulate production of CXCL9 and CXCL10 upon encountering the vector, with particularly for the latter chemokine a possible contribution from infected non-immune cells such as muscle cells (42, 43). These chemokine signals activate the CXCR3 receptor present on effector and memory CD8 T cells, instigating CD8 T cell priming and differentiation, and tissue infiltration of these cells due to chemotaxis along the CXCL9/CXCL10 gradient. The subsequent antigen recognition, due to their interaction with either infected immune or muscle cells, or with cells which phagocytosed an infected cell or viral particle, triggers these T cells to produce IFN-γ. Of note, IFN-γ–expressing T cell responses to ChAd155-RG were previously detected in spleens from immunized mice, and in peripheral blood from the immunized macaques described here (25). Possibly aligned, the NF-kB-associated pathway, linking to both innate and adaptive regulation, and the adaptive immunity-related pathway both appeared to be more upregulated in blood at D7 vs. D3.

Interestingly, similar kinetics, with a second cytokine peak at D7, was seen in serum from NHP injected with a comparable dose of the (Group D) Ad48 vector, but not with Ad5, Ad26, or Ad35 vectors (20), suggesting that this pattern may be vector serotype-specific. Since Ad48, Ad26 and Ad35 utilize the CD46 receptor, while Ad5 and ChAd155 [which has high genome similarity with Ad5 (12)] supposedly use mainly CAR, these data suggest that the involved immune determinants are not limited to receptor usage. For Ad48, it has been proposed that this bimodal pattern was specific to its hexon hypervariable region, and that in addition to fiber-receptor interactions, other Ad capsid components could also induce innate immune responses (20). This hypothesis may warrant further investigation for ChAd155.

In both models, the vector elicited only low IFN-α and IFN-γ responses relative to baseline/control, as seen for Ad5 in monkeys (20), but also clear IFN-associated response patterns, comprising increased CXCL9/10 levels and IFN-related gene expression in blood and/or tissues. For another (subcutaneously delivered) C-serotype ChAd vector, IFN/STING signaling-related gene expression had a negative impact on the CD8^+^ T-cell kinetics, which was mediated, amongst others, by suppressed antigen translation in DCs (17). We detected local transgene expression in both tissues, with DNA levels in muscle up to D14 possibly representing a reservoir that is necessary to mount adaptive immunity. In addition, in both animal models, the presented (**Figure S2**) or published (25) antibody or T-cell data suggest that at least for this vector, IFN-associated responses and robust antigen-specific adaptive immunity can co-exist. It should be noted however that the conclusion of an IFN/STING-mediated impact on adaptive immunity drawn in the former study (17), was mainly based on gene expression in murine blood and dLNs, without supporting characterization of local cytokines and immune cell infiltration. Comparison of our data with that study is also obfuscated by differences in the delivery method, which is known to affect vaccine responses (19), and in the number of analysis time points. Finally, the same study, as well as a study using intramuscularly injected vectors, also revealed vector group-dependent differences in IFN expression and transgene expression (17, 44), with a tendency toward higher levels of transgene expression for Group E vectors as compared to Group C vectors. Altogether, further mechanistic research is needed to investigate the interplay between innate and adaptive immunity for ChAd155-vectors and vectors from other groups, which could be guided by the current observations.

Finally, we note that i.m.-delivered ChAd155 vectors did not appear to elicit substantial inflammatory responses. This considering our data obtained for murine tissues—low-level neutrophil or eosinophil infiltration, negligible CXCL1, CSF-3, or IL-6 responses relative to controls―and for NHP blood, i.e., decreased IL-8 and increased IL1Ra levels relative to baseline. This may be of interest since blood cytokine profiles can be Ad serotype-specific, given the pro-inflammatory cytokine (IL-6, IL-1β, TNF-α) responses seen in NHP with (Group B/D) Ad35 or Ad48 vectors, but not with an Ad5 vector (20). Still, whether our data can be linked to either the absence of clinical symptoms in ChAd155-RG-injected NHP (25) or the low-grade reactogenicity of ChAd155-RSV in humans (7), remains to be elucidated.

We conclude that the observed response dynamics was consistent across most of the read-outs and seen in both animal models. Further investigation using the i.m. route, e.g., by confocal analysis of both the injected muscle and the dLN after ChAd-GFP injection, is still warranted. This may help to (i) link these early responses to the adaptive immunity induced by these ChAd-vectored vaccines, (ii) extend the current blood transcriptomic data to local gene expression in NHP, and (iii) determine to what extent our data can be translated to other serotypes, vector doses and host immune systems, which may be particularly relevant for recently developed adeno-vectored candidate vaccines against COVID-19 (45, 46). Another critical aspect with respect to the latter and other Ad-based vaccines is the boostability of the response. Indeed, in macaques, adaptive responses to the first dose of ChAd155-RG appeared to be boostable by a second dose administered 48 weeks later (25). Boostability was also observed for SARS-CoV-2 vaccines based on ChAdOx1, in mice and pigs (47) as well as in a Phase1/2 trial in humans (48). However, it was inconsistently observed across read-outs in human ChAd155-RSV vaccinees, possibly due to the presence of baseline anti-vector responses in some subjects (7). Collectively these studies demonstrate that multiple determinants are at play, such as vector identity and dose, the time interval between doses, and the immune status of the host.

Nonetheless, we show for the first time that the local innate immunity to i.m. injected ChAd155 vector mostly followed a distinctly bimodal kinetic pattern with tissue-specific response characteristics, and a propensity toward IFN pathway-associated responses. These findings represent promising avenues for future research, which may facilitate design of novel ChAd-based vaccines, and could ultimately advance the adenoviral vaccine platform at large.

## Data Availability Statement

NanoString gene expression data has been made available through Supplementary data in manuscript. Additional data can be made available upon request to corresponding author.

## Ethics Statement

The animal study was reviewed and approved by AAALAC-accredited facilities of GSK (Rixensart, Belgium) and Aptuit (Verona, Italy). Animal husbandry and experiments were ethically reviewed and carried out in accordance with the European Directive 2010/63/EU and the GlaxoSmithKline Biologicals S.A. Policy on the Care, Welfare and Treatment of animals. The study in NHP was conducted in accordance with the Italian legislation, under approval of the facility’s Committee on Animal Research and Ethics, and under authorization issued by the Italian Ministry of Health (Italian Ministry of Health Authorization n. 984/2015-PR; Aptuit Internal code no. 50000).

## Author Contributions

CC and AC were involved in the conception and design of the studies and/or the development of study protocols. CC, AC, and VBo participated to the acquisition of data. CC, AC, and VBo analyzed and interpreted the results. All authors contributed to the article and approved the submitted version.

## Funding

This work was sponsored by GlaxoSmithKline Biologicals SA, which was involved in all stages of the study conduct and analysis and took responsibility for all costs incurred in publishing.

## Conflict of Interest

CC, VBo, AC, NS, SM, RB, VBe, and ST are, or were at the time of the study, employees of the GSK group of companies. RB, SM, VBe, and ST report ownership of GSK shares and/or restricted shares. SC is an employee of Reithera Srl.
