# Supplementary material for: Innate Immune Responses to Chimpanzee Adenovirus Vector 155 Vaccination in Mice and Monkeys
Source: Front Immunol. 2020 Nov 30;11:579872. doi: 10.3389/fimmu.2020.579872 (PMC7734297; doi:10.3389/fimmu.2020.579872)
Supplement: Supplementary file 1 [file DataSheet_1.docx]

Supplementary Material

# Supplementary Methods: Analysis of raw whole blood gene expression data in NHP using Nanostring

Raw data was analyzed using Nanostring nSolver 4.0 software and Advanced Analysis Module 2.0 plugin, as described (1,2). Standard count normalization was performed as follows. We first calculated a global background (using the determined threshold count of 20). To adjust for system variation between lanes, we then normalized to internal positive controls by calculating the positive control factor (mean of geometric means of positive controls of the different lanes), and for each lane, a normalization factor (positive control factor divided by the geometric mean of each lane) was used as a multiplier for the lane’s count values. To identify genes suitable for use as input normalization factors, all genes were filtered to include only genes with count values exceeding the global background in all samples. Then the geNorm algorithm (1) in Nanostring nSolver was used, and the 10 top-ranking most stable genes were selected, which were then subjected to reference gene normalization across the samples. Gene ratios (log_2_) were used for differentially expressed genes (DEGs) and gene-set enrichment analyses, using the Nanostring nSolver Advanced Analysis Module 2.0. Multivariate differential expression analysis was performed through the optimal method for each gene, using a negative binomial mixture model for low expression probes, or a simplified negative binomial model for high expression probes, by defining timepoint as predictor and sample as confounder. Derived p-values were corrected for multiple testing using Benjamini-Hochberg’s method (2). To facilitate the biological interpretation, two pathway analysis methods were used: the pathway scoring module and the gene-set analysis module. Further data analysis was performed as described in Materials & Methods ‘*Whole blood gene expression in NHP using Nanostring*’.

**References**

1. Vandesompele J, De Preter K, Pattyn F, Poppe B, Van Roy N, De Paepe A, et al. Accurate normalization of real-time quantitative RT-PCR data by geometric averaging of multiple internal control genes. *Genome Biol.* (2002) 3:RESEARCH0034. doi: 10.1186/gb-2002-3-7-research0034

2. Wang H, Horbinski C, Wu H, Liu Y, Sheng S, Liu J, et al. NanoStringDiff: a novel statistical method for differential expression analysis based on NanoString nCounter data. *Nucleic Acids Res.* (2016) 44:e151. doi: 10.1093/nar/gkw677

# Supplementary Tables

## Supplementary Table 3. Upregulated genes in ChAd155-RG-treated macaques analyzed by qPCR

| Gene | Day 1 | Day 3 | Day 7 |
| --- | --- | --- | --- |
| CASP1 | **1.40** | -0.70 | 0.62 |
| ***CXCL10*** | **3.45** | **1.26** | **2.56** |
| ***DDX58*** | **3.19** | -0.75 | **1.21** |
| ***ICAM1*** | **1.08** | 0.06 | 0.14 |
| ***IRF7*** | **2.39** | -0.11 | **1.70** |
| ***JAK2*** | **1.20** | -0.01 | 0.73 |
| ***LYZ*** | **1.00** | 0.49 | 0.73 |
| ***MX1*** | **2.55** | -0.74 | **1.14** |
| ***STAT1*** | **1.68** | -0.16 | 0.98 |
| TLR3 | **1.72** | 0.37 | 0.47 |
| ***TLR7*** | **1.27** | 0.09 | -0.07 |
| IL4 | -0.27 | 0.46 | **1.22** |

Gene expression levels determined in whole blood samples from macaques immunized with ChAd155-RG were measured by qPCR. N=6/time-point. Genes differentially expressed over prevaccination on at least one time-point are shown. Data are presented as mean fold changes over baseline (in log_2_), with values >1 or < -1 as considered over- or under-expressed, respectively. Names of IFN-related genes are highlighted in bold italic font. Upregulated values are highlighted in bold font.

# Supplementary Figures

**Supplementary Figure 1.** **Common gating strategy for flow cytometry analysis of immune cell subsets in murine tissues.** Total events were gated based on singlets and FSC/SSC, excluding dead (Live/Dead-positive) cells and auto-fluorescent cells. The gating strategy allowed identifying the main populations (B cells: CD19^+^ MHCII^+^ cells; T cells: NK1.1^-^ TCRβ^+^ cells), as well as the innate immune-cell subsets infiltrating the tissues, comprising neutrophils (SSChiCD11b^+^Ly6G^+^ cells), eosinophils (SSC^hi^ SiglecF^+^ cells), granulocytes (SSC^hi^ Ly6C^-^ MHCII^-^ CD11b^+^ cells), total dendritic cells (DCs: plasmacytoid DCs [pDCs: CD11b^-^ SiglecH^+^ cells], resident DCs [CD11c^hi^ MHCII^mid^ cells] and migratory DCs [CD11b^+/-^ CD11c^+^ MHCII^hi^ cells]), monocytes (CD11b^+^ Ly6C^+^ cells; identified after excluding DC subsets), and the total NK-cell population (SSC^lo^ Ly6C^-^ CD11b^-^ CD11c^-^ cells: NKT [NK1.1^+^ TCRβ^+^] and NK [NK1.1^+^ TCRβ^-^] cells). Antibody panels are further detailed in the Materials and Methods. The gating strategy presented below was used in the experiments in draining lymph nodes, including those using green fluorescent protein (GFP); see Main Figures 3 and 4B. In the experiments identifying muscle-infiltrating immune cells (see Main Figure 3), a similar gating strategy was used but with the following changes: (1) CD45 was used as marker to discriminate hematopoietic cells from muscle cells; (2) NK cells were detected using NKp46; and (3) monocytes were identified as CD11b^+^ cells expressing low levels of Ly6C and F4/80; in the experiments detecting GFP in the muscles (see Main Figure 4B), cells were only stained with the CD45 marker.

**Figure S2. Antibody response to ChAd155-RSV in mice.** C57BL/6 mice received a single intramuscular injection of ChAd155-RSV (10^8^ viral particles/animal) at Day 0. Anti-RSV F immunoglobulin (Ig)G concentrations were measured in sera using ELISA with a cut-off of 50 ELISA units (EU)/mL. Data from two separate experiments are shown. Blood samples were collected daily from Day 1 through Day 14 post-vaccination in the first experiment (n=5/time-point; circles), and at Days 14, 21, 34, 56 and 77 post-vaccination in the second experiment (n=10/time-point; squares). Data are presented as IgG concentrations per animal (open symbols) and geometric mean concentrations per time-point (lines and filled symbols).

**Supplementary Figure 3.** **Selected cytokine responses in murine tissues and serum.** Cytokine responses in the injected muscle and draining lymph node (dLN) (**A**) and serum (**B**) collected from mice treated with ChAd155-RSV (10^8^ vp) or buffer (‘Control’) at Day 0 are shown (n=5 per group per timepoint). Injections (10 µL) were performed unilaterally in the left gastrocnemius muscle at Day 0. Tissues were collected before immunization (0 h), and after immunization at 1 h, 3 h and 6 h, and then daily from 24 h (Day 1) through Day 14. Graphs represent data from a single experiment. Cytokine levels (expressed in pg/organ) were detected in homogenate supernatants of the indicated tissues collected from the injected side of each animal. Geometric mean concentrations are presented as lines (**A**, **B bottom**), or cumulative bars (**B top**). Each symbol in the line graphs represents an individual animal.
